# Common and rare variants in complement genes as biomarkers of COVID-19 infection and severity. A lesson to learn for emerging pathogens

**DOI:** 10.3389/fimmu.2026.1829115

**Published:** 2026-06-11

**Authors:** María Eugenia De La Morena-Barrio, Ana Van Den Rym, Olga Escorial Sanz, Fernando Corvillo, Rosario García-Sánchez, Laura González-Sánchez, Adrián Muñoz-Barrera, Rafaela González-Montelongo, José Miguel Lorenzo-Salazar, Carlos Flores, Ana de Andrés-Martín, Carlos Rodríguez-Gallego, Luis Allende, Laia Alsina, Silvia Sánchez-Ramón, Eduardo López-Collazo, Margarita López-Trascasa, Pilar Sánchez-Corral, Rebeca Pérez de Diego, Javier Corral de la Calle, Alberto López-Lera

**Affiliations:** 1 Servicio de Hematología, Hospital Universitario Morales Meseguer, Centro Regional de Hemodonación, Universidad de Murcia, Instituto Murciano de Investigación Biosanitaria (IMIB)-Arrixaca, Murcia, Spain; 2 Centro de Investigación Biomédica En Red (CIBER) de Enfermedades Raras (CIBERER), Instituto de Salud Carlos III, Madrid, Spain; 3 Interdepartmental Group of Immunodeficiencies, Madrid, Spain; 4 Laboratory of Immunogenetics of Human Diseases, IdiPAZ Institute for Health Research, La Paz University Hospital, Madrid, Spain; 5 Innate Immunity Group, IdiPAZ Institute for Health Research, La Paz University Hospital, Madrid, Spain; 6 Emergencias SUMMA112, Madrid, Spain; 7 Complement Research Group, IdiPAZ Institute for Health Research, La Paz University Hospital, Madrid, Spain; 8 Genomics Division, Instituto Tecnológico y de Energías Renovables (ITER), Santa Cruz de Tenerife, Spain; 9 Research Unit, Hospital Universitario Ntra. Sra. de Candelaria, Instituto de Investigación Sanitaria de Canarias (IISC), Santa Cruz de Tenerife, Spain; 10 CIBER de Enfermedades Respiratorias (CIBERES), Instituto de Salud Carlos III, Madrid, Spain; 11 Department of Clinical Sciences, University Fernando Pessoa Canarias, Las Palmas de Gran Canaria, Spain; 12 Department of Immunology, Ramón y Cajal Hospital, Madrid, Spain; 13 Department of Immunology, University Hospital of Gran Canaria Dr. Negrin, Las Palmas de Gran Canaria, Spain; 14 Instituto de Investigación Sanitaria de Canarias, Las Palmas de Gran Canaria, Spain; 15 Department of Medical and Surgical Sciences, School of Medicine, University of Las Palmas de Gran Canaria, Las Palmas de Gran Canaria, Spain; 16 Department of Immunology, 12 de Octubre Hospital, Madrid, Spain; 17 Clinical Immunology and Primary Immunodeficiencies Unit, Pediatric Allergy and Clinical Immunology Department, Hospital Sant Joan de Déu, Barcelona, Spain; 18 Institut de Recerca Sant Joan de Déu, Barcelona, Spain; 19 Clinical Immunology Unit, Hospital Sant Joan de Déu-Hospital Clínic Barcelona, Barcelona, Spain; 20 Clinical Immunology Department, San Carlos Clinical Hospital, Madrid, Spain; 21 UNIE University, Madrid, Spain; 22 Departamento de Medicina, Universidad Autónoma de Madrid, Madrid, Spain

**Keywords:** ARDS (acute respiratory disease syndrome), complement system, COVID- 19, genetic variation, innate immunity

## Abstract

**Introduction:**

The first wave of Coronavirus disease 2019 (COVID-19), driven by the global emergence of Severe Acute Respiratory Syndrome Coronavirus 2 (SARS-CoV-2), severely affected Spain with high infection and mortality rates across the country. Although numerous common and rare genetic variants affecting immune-related pathways have been associated with susceptibility to infection and severe disease, the contribution of complement system remains comparatively understudied.

**Methods:**

In this work, we analyzed the frequencies and severity associations of complotype-related common polymorphisms and rare complement variants in whole-exome sequencing data from a Spanish cohort accounting for 154 adults hospitalized due to severe COVID-19.

**Results:**

Our results indicate that the *CFHR4* rs7417769 (p.N209S) and CFH *rs1061170* (p. Y402H) common polymorphisms are significantly associated with protection against acute respiratory distress syndrome (ARDS), while the *C3* rs2230199 (p.R102G) and *MASP2* rs7255087 (p.D120G) polymorphisms respectively correlated with low and high *C3* levels. The marked over-representation of the *C1R* rs117402032 and *C8A* rs143523574 polymorphisms and increased frequency of heterozygous carriers of alleles previously associated with low FCN2 and FCN3 levels, suggest a link beween defective complement activation and increased rates of SARS-CoV-2 infection and support a pivotal role for the lectin pathway in the pathogenesis of COVID-19.

**Discussion:**

Together, these results demonstrate that common variants in complement genes modulate susceptibility to severe COVID-19 and its clinical complications. They also identify promising, testable genetic biomarkers with potential utility not only for SARS-CoV-2, but also for preparedness against future emerging infectious threats.

## 1 Introduction

Coronavirus disease 2019 (COVID-19), due to the infection by Severe Acute Respiratory Syndrome Coronavirus 2 (SARS-CoV-2), caused a global pandemic starting in December 2019. With human‐to‐human transmission as its main epidemic mechanism and in-hospital mortality rates of approximately 11%, increasing to 21% in individuals above 75 years old, COVID-19 represents the most significant global public health challenge of the 21st century (1). The course of COVID-19 infection varies from mild to the life‐threatening acute respiratory distress syndrome (ARDS), sepsis and multiorgan failure. Severity and outcome of COVID-19 critically depend on the complex interaction of a variety of internal and external factors. Environmental components, viral properties, and the physiological characteristics of the host contribute to dealing with the pathogen. In this regard, both the innate and adaptive arms of the immune system have been the focus of extensive studies, with polymorphic variants affecting critical components of immunity being identified as either risk or protective factors that modify the course of COVID-19 infection (2, 3). As an ancient part of innate immunity, the complement system is critical in the protection of the host against infection by viral pathogens (4, 5). Moreover, it interfaces with adaptive immunity, helping eliminate viruses and virus-infected cells through a multitude of mechanisms, including the opsonization of viruses and virus-infected cells, induction of inflammatory states and modulation of late adaptive responses (6, 7).

In the context of COVID-19 disease, immunological studies have led to a current understanding of the bipolar role of the innate immune response (8, 9). While a strong initial complement response seems to provide beneficial effects in the prevention of viral entry and fighting against the dissemination of the pathogen, its overactivation in later stages of the disease has been linked to detrimental effects and can lead to ARDS and the Multisystem Inflammation Syndrome (MIS) (10, 11). Complement activation by SARS-CoV-2 takes place through its three (classical, alternative and lectin) pathways and it is a usual feature in ARDS, with elevated C5a levels being a proposed biomarker of the condition (12). Complement can be directly activated by the SARS-CoV-2 S- and N-proteins through the binding of the lectin pathway recognition molecules MBL, FCN2 and CL11. These interactions result in C4b and C3b generation, cellular deposition, and C3 convertase assembly (13). The complement system is also activated in the alveolar epithelia and pulmonary vascular endothelia of COVID-infected individuals via the alternative pathway, and produced locally by respiratory epithelial cells following infection (14). Alveolar macrophages synthesize complement proteins and respond to complement activation through the expression of CR1, CR3, CR4, C3aR and C5aR1 complement receptors (15). From 2020 onwards, several studies have suggested that common genetic variation in complement proteins may influence the course and severity of COVID-19 infection. This includes single nucleotide polymorphisms affecting components of the classical, alternative and lectin-mediated pathways (16, 17).

In this report, we have analyzed complement genetic variation in whole-exome sequencing (WES) data from 154 unrelated adult patients of European descent hospitalized due to severe COVID-19 disease during the first wave of the pandemic in 2020.

## 2 Materials and methods

### 2.1 Study cohort and biological samples

For the present work, two subcohorts of COVID-19 unrelated Spanish individuals infected during the first wave of the pandemic were analyzed:

- Subcohort 1 included WES data from 103 adult Spanish patients of Non-Finnish European (NFE) descent requiring hospital admission at Morales Meseguer and Reina Sofia University Hospitals (Murcia, Spain) from March to April 2020. Exclusion criteria included chronic oral anticoagulation at the time of blood sampling and patients younger than 18 years of age. The severity and disease course of the patients included was variable, ranging from mild to critical (ARDS-associated). Biochemical and clinical data were available from 87 patients of this cohort. Subcohort 1 study was approved in the administrative resolution EST 24/20 by CEI-CEIm Hospital General Universitario José María Morales Meseguer.

- Subcohort 2 was composed of WES data from 51 adult Spanish patients of NFE descent with critical COVID-19 all of whom were admitted to the ICU and had a diagnosis of ARDS at different Spanish hospitals: 12 de Octubre University Hospital (Madrid), Hospital Sant Joan de Déu (Barcelona), University Hospital of Gran Canaria Dr. Negrin, (Las Palmas de Gran Canaria), University Hospital La Paz (Madrid), Hospital Clínico San Carlos (Madrid), and Hospital Universitario Ramón y Cajal (Madrid). The DNA for WES was obtained during May/September 2020.

In both subcohorts, SARS-CoV-2 infection was assessed by quantitative real-time reverse-transcriptase polymerase chain reaction (qRT-PCR) at the time of hospital admission using the diagnostic criteria defined in the World Health Organization (WHO) interim guidance22.

The study was conducted according to the Declaration of Helsinki and approved by the Clinical Research Ethics Committee of La Paz University Hospital. All participants provided written informed consent for participation in the study and for the publication of their clinical and biochemical information.

### 2.2 Human molecular genetics and whole-exome sequencing

In subcohort 1, genomic DNA was extracted from peripheral whole blood samples from the subjects using the QIAamp DNA Blood Mini kit (Qiagen GmbH, Hilden, Germany), according to manufacturer’s protocol.

Whole-exome sequencing analysis was performed by Beijing Novogene Bioinformatics Technology. Briefly, human genomic DNA was sheared mechanically and prepared as libraries containing duel-indexed sequencing barcodes. The pre-capture libraries were enriched using SureSelect Human AII Exon V6 capture baits (Agilent Technologies) for coding regions and splice junction sites of 20,000 human genes. The post-capture libraries were sequenced on a NovaSeq 6000 instrument (Illumina Inc.). The sequence data were analyzed using a custom-developed bioinformatics pipeline which aligns sequence data to human genome (GRCh37/UCSC hg19) and performs small variant calling and annotation. The pipeline also performed QC analysis of the sequence data to ensure that the reported variant findings were obtained from quality sequencing data.

In subcohort 2, whole-exome sequencing was performed by the New York Genome Center with an Illumina HiSeq 2500 machine and an Agilent 71Mb SureSelect exome kit. The reads were aligned with the human reference genome (GRCh37/UCSC hg19) with a BWA aligner (18), then recalibrated and annotated with GATK (19), PICARD (http://picard.sourceforge.net/) and ANNOVAR (20). The variants were then filtered and investigated with an in-house online server.

### 2.3 Clinical and biochemical data collection

The clinical data collected for multivariate analysis included age, sex, smoking status, arterial thrombosis, diabetes mellitus, dyslipidemia, Charlson score, Sepsis-related Organ Failure Assessment (SOFA) and quick SOFA scores, CURB-65 (confusion, uremia, respiratory rate, BP, age ≥ 65 years) score, cardiovascular disease, chronic obstructive pulmonary disease, asthma, hepatopathy, chronic kidney disease, cancer, immune suppression, angiotensin II receptor blockers, angiotensin converting enzyme inhibitors, non-steroidal anti-inflammatory drugs, ARDS, and ICU admission.

The biochemical and cellular data analyzed were: serum levels of C3, C4, creatinine, glucose, calcium, creatinine phosphokinase (CPK), glutamic-oxaloacetic transaminase (GOT), glutamic-pyruvic transaminase (GPT), bilirubin, lactate dehydrogenase (LDH), hemoglobin, platelets, D-dimer, C reactive protein (CRP), procalcitonin (PCT), interleukin-6 (IL6), ferritin, leukocyte numbers, lymphocyte numbers, neutrophil/lymphocyte ratio, human immune-deficiency virus (HIV), hepatitis B virus (HBV), hepatitis C virus (HCV), ABO and Rh blood typing, sedimentation rate, serum immunoglobulins IgG, IgA, and IgM,

### 2.4 Statistical analysis

Statistical analysis was performed with GraphPad Prism version 10.6.1 (GraphPad Software, Boston, Massachusetts USA). Clinical data were analyzed by multiple linear regression by the least squares method, Kolmogorov-Smirnov normality test and no specific weighting method. Once identified, significative correlations were further analyzed: the effects of specific complement polymorphisms on the levels of complement proteins C3 and C4 in serum were analyzed by Unpaired t-test when only two groups were considered (wild type versus heterozygous patients) or by One-way ANOVA with Bonferroni correction for multiple comparison tests when more than two groups (major allele homozygous, heterozygous and minor allele homozygous) were included in the analysis (CFH rs1061170 and *CFHR4* rs7417769). Differences were deemed statistically significant at *p ≤* 0.05.

As a cohort observational study in the absence of case/control analysis, the estimates of ARDS risk were calculated as risk ratios (RR) (RR= ARDS risk of minor allele carriers/ARDS risk of major allele homozygotes). Contingency analysis was performed with the Fisher extract test followed by p value adjustment with the Holm-Sidak method for multiple comparisons. The 95% confidence intervals (CI) were calculated using the Koopman asymptotic score.

## 3 Results

### 3.1 Common complement variation in COVID-19 patients

We compared the incidence of four common SNPs affecting complement components with their reported allele frequencies (AF) in the Non-Finnish European (NFE) population in gnomAD and Ensembl genome databases as of January 2025: *C3* rs2230199 (c.304C>G; p.R102G), *CFH* rs800292 (c.184G>A; p.V62I) and rs1061170 (c.1204T>C; p. Y402H) and *CFHR4* rs7417769 (c.626G>A; p.N209S) (**Table 1**). The selected polymorphic variants have been previously associated with either altered complement levels and/or risk of disease in humans, are representative of common variation in complement function and segregate with frequencies between 20% and 38% in NFE populations. Two of these variants (*CFH* rs800292 (p. V62I) and *C3* rs2230199 (p.R102G)) are part of the “complotype” group of common polymorphisms linked to major effects (up to sixfold variation) in complement hemolytic activity *in vitro* (21). The *C3* rs2230199 minor (c.304G) and *CFH* rs800292 major (c.184G) alleles have been associated with high complement activation rates, reduced risk of infection and increased risk of inflammation. However, each of these variants has a minor effect, and the combined inheritance of several variants is required to see a statistically significant disease association (18).

**Table 1:** Common complement variation in COVID-19 patients.

	rs2230199 *C3* (c.304C>G) p.R102G	rs1061170 *CFH* (c.1204T>C) p.Y402H	rs800292 *CFH* (c.184G>A) p.V62I	rs7417769 *CFHR4* (c.629G>A) p.S209N
Number of cases				
Major allele homozygotes	113	50	68	54
Heterozygotes	36	60	67	54
Minor allele homozygotes	5	44	19	46
Frequency				
Minor allele	0.15	0.48	0.341	0.474
European (Non Finnish)	0.2133	0.3836	0.2301	0.3018
Frequency Ratio	0.706	1,251	1,482	1,571
NON-ARDS				
Number of cases				
Major allele homozygotes	30	5	20	4
Heterozygotes	16	18	21	24
Minor allele homozygotes	2	24	7	20
Frequency				
Minor allele	0.208	0.702	0.314	0.666
Frequency Ratio	0.975	1.83	1,365	2,207
ARDS				
Number of cases				
Major allele homozygotes	62	32	34	38
Heterozygotes	13	39	34	23
Minor allele homozygotes	2	19	8	14
Frequency				
Minor allele	0.11	0.427	0.328	0.34
Frequency Ratio	0.516	1,113	1,425	1,127
ARDS RR (95% CI)	0.62 [0.41-0.99]	0.32 [0.14 to 0.69]	0.93 [0.58-1.44]	0.18 [0.07 to 0.42]
Adjusted p value	0.2	0.0146	0.853	5.06x10^-6^

(A) Number of patients carrying the selected common complement variants in the heterozygous or homozygous condition in the global COVID-19 cohort. The observed frequencies and expected incidence of the minor alleles in Non-Finnish Europeans (using gnomAD data in v.4.1.0 release) were used to calculate variant-specific frequency ratios (observed/expected). (B) Distribution of variants frequencies in ARDS and non-ARDS patients, frequency ratios and risk ratios for ARDS (ARDS-RR). Contingency analysis was performed with the Fisher extact test followed by p value adjustment with the Holm-Sidak method for multiple comparisons. The 95% confidence intervals (CI) were calculated using the Koopman asymptotic score. Bold values indicate statistically significant associations.

**Figure 1:**
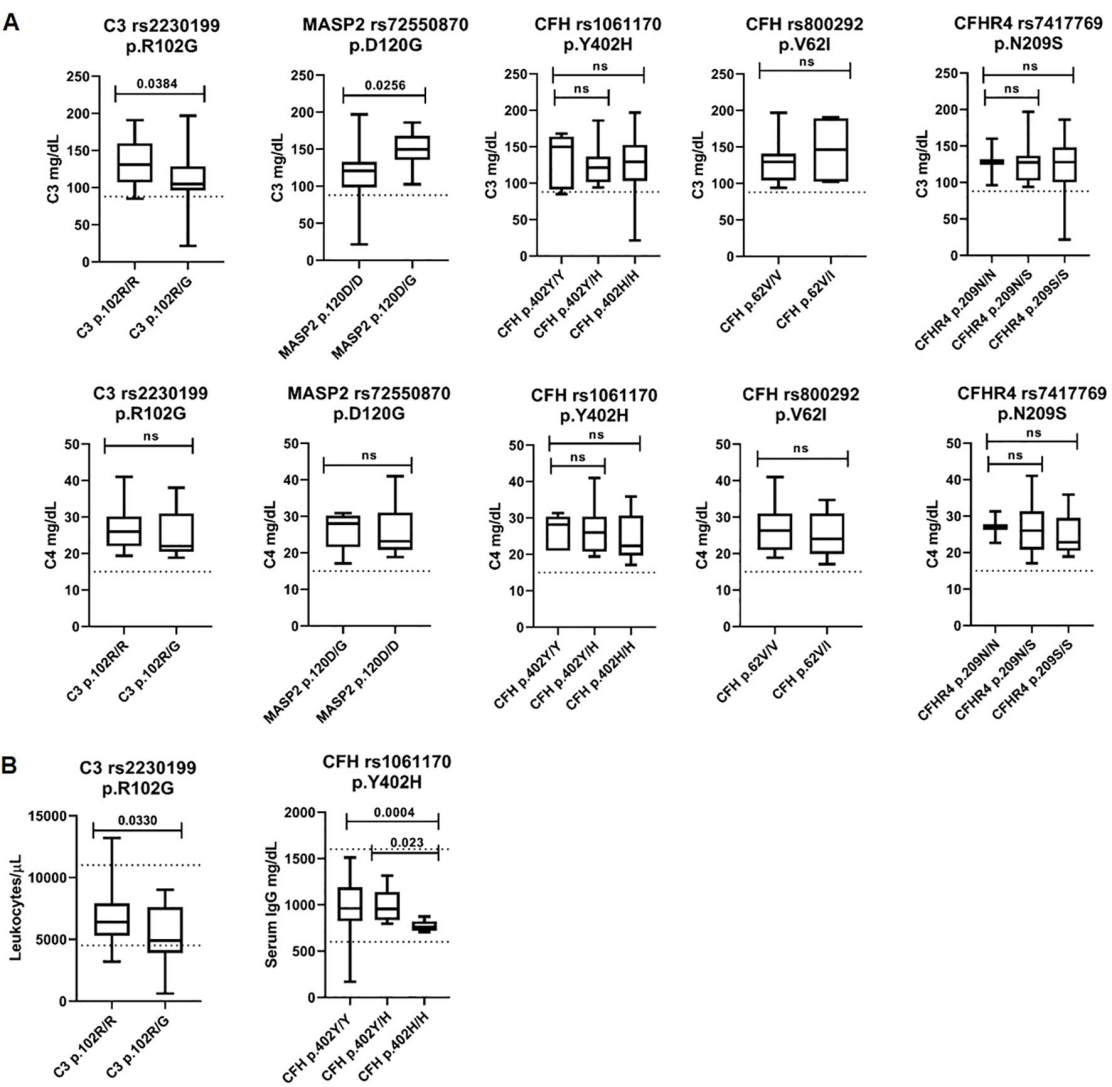
Correlation of complement variants with complement levels and cellular biomarkers. **(A)** The correlations of each complement variant with the serum levels of C3 and C4 are shown. In each box and whiskers plot, the leftmost group corresponds to major allele homozygotes and the groups to the right denote heterozygous and, when applicable, homozygous carriers of the minor allele. For the *MASP2* rs7255087 (Asp120Gly), *C3* rs2230199 (Arg102Gly) and *CFH* rs800292 (Val62Ile) variants, the low number of minor allele homozygotes prevented their inclusion in the statistical analysis and only heterozygous cases are considered. The high population frequency of the *CFH* rs1061170 (Tyr402His) and *CFHR4* rs7417769 (Asn209Ser) variants allowed the inclusion of minor allele homozygotes. Heterozygous carriers of the rs7255087 variant in *MASP2* showed significantly higher C3 levels (p=0.0256) while those carrying the rs2230199 variant in *C3* presented with significantly lower C3 values (p=0.0384). The lower limit of each biomarker’s normal range is indicated by dotted lines in each plot. **(B)** The *C3* rs2230199 (Arg102Gly) variant correlated with leukocyte counts in plasma samples (p=0.033) while the homozygotes for the *CFH* rs1061170 (Tyr402His) variant showed lower IgG levels in serum compared to heterozygous (p=0.023) and wildtype counterparts (p=0.0004). Normal ranges are indicated by dotted lines in each plot.

The *C3* rs2230199 (c.304C>G; p.R102G) variant has been extensively scrutinized in disease-association studies. As a “high activation” complotype variant, it is associated with high hemolytic activity *in vitro* and increased risk of age-related macular degeneration (AMD) (21). It is responsible for the electrophoretic variants “Slow” (known as C3S and carrying an arginine residue) and “Fast” (known as C3F and carrying a glycine). At an observed AF of 15% in our COVID-19 cohort, its minor allele was under-represented as compared to its expected population frequency (0.7xNFE) with differences between the ARDS (11%) and non-ARDF (20.8%) subcohorts. Thus, it was associated with a non-significant tendency to protection toward ARDS (ARDS-RR= 0.62 [95% CI: 0.41-0.99] adjusted *p* = 0.2) and showed a significant association with low C3 levels (104.4 mg/dL in c.304G carriers versus 131.1 mg/dL in c.304C controls; *p* = 0.038) in COVID-19 patients. Moreover, multivariate analysis and Mann-Whitney U-test showed that the variant correlated with decreased leukocyte counts in plasma (*p* = 0.033) (**Figure 1**).

Two common variants affecting the *CFH/CFHR* locus were associated with significant effects on the ARDS-RR in the cohort:. The rs1061170 (c.1204T>C; Y402H) variant in the *CFH* gene is strongly associated with both dry and wet forms of AMD (22, 23) and its minor (C) allele has been associated with reduced regulation and increased complement activation in some cohorts (24). It was found at an AF of 48% (1.25x NFE) in the COVID-19 cohort, which increased to 70% (1.83x NFE) in the case of non-ARDS patients and significantly associates with a protective ARDS-RR of 0.32 [0.14 to 0.69] (*p* = 0.0146**) (****Table 1**). Moreover, homozygous carriers of the variant presented with significantly lower serum IgG levels (**Figure 1**).

The rs7417769 (c.626G>A; p.N209S) in the *CFHR4* gene is a common variant found in around 30% of NFE. It has been previously associated with reduced FHR4 levels (p = 2.2 × 10^-56^) and a very significant protective effect toward AMD (25, 26). It was detected in 54 heterozygous and 46 homozygous patients in our cohort, which represents a relative enrichment of 1.57x in COVID-19 patients. Further clustering according to ARDS revealed a marked over-representation in the non-ARDS subcohort (66%) (ARDS-RR=0.18 [0.07 to 0.42] *p=*5.06x10^-6^). All considered, our data suggest that the rs7417769 variant substantially decreases the chances of progressing to ARDS in COVID-19 patients.

Interestingly, from 56 patients simultaneously carrying the *CFH* rs1061170 and *CFHR4* rs7417769 variants, 39 (69.6%) did not develop ARDS. The high correspondence between these associations cannot be attributed to linkage disequilibrium effects inside the *CFH/CFHR* locus, as rs1061170 and rs7417769 are poorly correlated in the Spanish population (r^2^ = 0.13; according to the Ensemble Genomic Browser) but instead suggest independent, cumulative effects. Certainly, the small number of co-segregation observations hinders any statistical analysis but suggest that polygenic haplotypes that include several of these variants may result in strongly protective phenotypes.

### 3.2 Rare polymorphic variants in complement genes

Due to the relatively small size of the cohort and to avoid spurious associations, for the analysis of rare (≤5% frequency) variants, a minimal representation threshold of the variants in 5% of the individuals was established. Based on this, seven polymorphic variants in genes encoding classical, alternative, lectin and terminal pathway complement proteins were selected (**Table 2**). Their expected and observed population frequencies, *in-silico* predictions and clinical associations are summarized in **Table 3**. With the exception of the *MASP2* rs72550870 variant, the scarcity of data regarding these variants prevented a minimally rigorous analysis of the associated clinical and biochemical data.

**Table 2:** Incidence of rare complement variants in COVID-19 patients.

	rs117402032 *C1R* (c.1030A>C) p.I359L	rs143523574 *C8A* (c.100C>T) p.P34S	*rs1620075 C8A (c.1454G>T) p.R485L*	*rs4151659 CFB (c.1693A>G) p.*K565E	rs72550870 MASP2 (c.359A>G) p.D120G	*rs532781899 FCN3 (c.316delC) p.L117S.fsX65*	*rs76267164 FCN2 (c.818G>A) p.R273Q*
Number of cases							
Major allele homozygotes	85	115	96	62	144	97	117
Heterozygotes	14	9	24	10	10	6	6
Minor allele homozygotes	0	2	0	0	0	0	0
Frequency							
Minor allele	0.0707	0.051587	0.1	0.06944	0.032	0.02912	0.02439
European (Non Finnish)	0.000204	0.0002806	0.04538	0.01274	0.03107	0.01504	0.00514
Frequency Ratio	347	184	2,204	5,451	1.03	1,936	4,745
NON-ARDS							
Number of cases							
Major allele homozygotes	41	47	32	42	43	45	47
Heterozygotes	4	1	11	7	5	4	2
Minor allele homozygotes	0	1	0	0	0	0	0
Frequency							
Minor allele	0.0444	0.03061	0.1279	0.0714	0.052	0.0408	0.02040
Frequency Ratio	218	109	2.81	5,604	1,673	2,713	3.97
ARDS							
Number of cases							
Major allele homozygotes	44	68	64	20	87	52	70
Heterozygotes	10	8	13	3	4	2	4
Minor allele homozygotes	0	1	0	0	0	0	0
Frequency							
Minor allele	0.09259	0.06493	0.08442	0.06521	0.0219	0.01851	0.02702
Frequency Ratio	454	231	1.86	5,119	0.705	1,231	5,258
ARDS RR (95% CI)	1.68 [0.83 - 4.21]	1.77 [0.78 - 5.09]	0.72 [0.425 - 1.14]	0.97 [0.69 - 1.74]	0.55 [0.35-1.10]	0.7 [0.46 - 1.58]	1.21 [0.54 - 4.21]
Adjusted p value	0.248	0.2475	0.3412	0.8870	0.4033	0.4203	>0.9999

Number of patients carrying the selected rare complement variants in the heterozygous or homozygous condition in ARDS and non-ARDS patients in the COVID-19 cohort. The observed frequencies and expected incidence of the minor alleles in Non-Finnish Europeans (using GnomAD data in v.4.1.0 release) were used to calculate variant-specific frequency ratios (observed/expected). Contingency analysis of the frequencies observed in ARDS versus non-ARDS patients was used to calculate ARDS risk ratios (ARDS-RR). Contingency analysis was performed with the Fisher extact test followed by p value adjustment with the Holm-Sidak method for multiple comparisons. The 95% confidence intervals (CI) were calculated using the Koopman asymptotic score.

**Table 3:** Genotype-phenotype correlations and *in silico*-predictions of rare complement variants in COVID-19 patients.

Variant	NFE	*In-silico* prediction					Associated phenotype	References
		SIFT	PolyPhen	CADD	REVEL	MEtaLR		
rs117402032 C1R (c.1030A>C) I359L	2.04 x10^-4^	0 (D)	0.003 (B)	5 (LB)	0 (LB)	0.004 (T)	NA	NA
*rs143523574 C8A (c.100C>T) P34S*	2.80 x10^-4^	0.04 (D)	0.169 (B)	11 (LB)	0.251 (LB)	0.017 (T)	NA	NA
*rs1620075 C8A (c.1454G>T) R485L*	4.54 x10^-2^	0 (D)	0.484 (PD)	12 (LB)	0 (LB)	0.002 (T)	NA	NA
*rs4151659 CFB (c.1693A>G) K565E*	1.27 x10^-2^	0.03 (D)	0.255 (B)	17 (LB)	0.091 (LB)	0.047 (T)	- Atypical hemolytic-uremic syndrome	- 27
							- Age-related macular degeneration	
*rs72550870 MASP2 (c.359A>G) p.D120G*	3.1 x10^-2^	0 (D)	0.982 (PD)	26 (LB)	0.762 (LDC)	0.103 (T)	- MASP2 deficiency	- 28
*rs532781899 FCN3 (c.316delC) L117S.fsX65*	1.50 x10^-2^	NA	NA	NA	NA	NA	- FCN3 deficiency	- 29
								- 30
*rs76267164 FCN2 (c.818G>A) R273Q*	5.14 x10^-3^	0.02 (D)	0.973 (PD)	22 (LB)	0.595 (LDC)	0.63 (D)	- Low FCN2 levels	- 31
							- Progressive multifocal leukoencephalopathy	- 32

*In-silico* predictions, population frequencies in Non-Finnish Europeans (NFE) and associated phenotypes available for the low frequency complement variants studied. The specific score of each algorithm (0 to 1) and the prediction of the variant’s impact on the protein phenotype (between parentheses) are shown. Legend: D, deleterious; PD, probably deleterious; LDC, likely disease-causing; LB, likely benign; T, tolerated; B, benign; NA, not available.

In this study, the main frequency deviations in relation to European population allele frequencies were found in the *C1R* rs117402032 and *C8A* rs143523574 polymorphisms. The rs117402032 (c.1030A>C: p.I359L) missense variant predicts a change of an isoleucine residue conserved through primates in the first SCR domain of C1r. It is an extremely rare variant in the European population, with an observed frequency of 0.02% albeit relatively common in Latin Americans (AF = 10.81%) and East Asians (AF = 2.82%). Overall, in our cohort, informative sequences of this SNP were available from 99 patients (54 with ARDS and 45 without ARDS). It was detected in 14 heterozygous patients of European descent, which represents an observed AF of 7.0%, with striking over-representation in both non-ARDS (218x NFE) and ARDS (454x NFE) patients (**Table 2**). No clinical associations have been previously reported for this variant. According to *in-silico* predictions, it is considered as benign or tolerated by CADD, PolyPhen, MetaLR, and REVEL algorithms but deleterious by SIFT (**Table 3**).

Two *C8A* variants with antagonistic clinical effects were over-represented in our cohort. The rs143523574 (c.100C>T p.P34S) variant, with a MAF of 0.028% in NFE, is classified as deleterious by SIFT and as benign or tolerated by PolyPhen, CADD and REVEL predictors (**Table 3**). It modifies the N-terminal domain of C8A by introducing a serine residue instead of the highly conserved proline at position p.34. In 126 available sequences spanning the polymorphism in the cohort (77 with ARDS and 49 without ARDS), it was extremely over-represented, being found in heterozygosis in 9 and in homozygosis in 2 patients for an observed AF of 5.1% (184x NFE). Moreover, this allele was overrepresented in ARDS vs non-ARDS patients (with frequencies of 6.5% vs 3.0%) (**Table 2**). The rs1620075 (c.1454G>T; R485L) variant is a comparatively more common polymorphism in Non-Finnish Europeans that affects a conserved arginine residue in the C-terminal portion of the Membrane Attack Complex/Perforin (MACPF) domain. With 24 heterozygous observed cases, the variant showed no frequency differences correlated with ARDS (ARDS-RR of 0.72 [95% CI: 0.425 - 1.14] (*p* = 0.3412)) (**Table 2**).

Other variants of interest were *CFB* rs4151659, MASP2 rs72550870, *FCN2* rs76267164 and *FCN3* rs532781899. *CFB* rs4151659 (c.1693A>G: p.K565E) is a missense variant reported in AMD cohorts but classified as benign by genomic databases (27) (**Table 3**). It was detected in heterozygosis in 10 patients out of a total number of 72 informative patients yielding an observed frequency of 6.9%. It is over-represented in both COVID-19 patients with ARDS (5.12x NFE) and without ARDS (5.6x NFE) (**Table 2**). The data are consistent with the rs4151659 being a risk factor for SARS-CoV-2 infection.

The MASP2 rs72550870 variant was detected in heterozygosis in 10 COVID-19 patients, which fits the expected population AF (3.11%) and suggests that it may not convey significantly increased susceptibility to SARS-CoV-2 and was not associated with ARDS in our study (ARDS-RR [95% CI]= 0.55 [0.35-1.10] p=0.4033) (**Table 2**). Notwithstanding the foregoing, the variant inversely correlated with complement levels during hospitalization: patients carrying the c.359G (Gly) allele had significantly higher C3 plasma levels than wild type c.359A (Asp) counterparts (142.8 vs. 120.7; p=0.0256) (**Figure 1**). C3 levels were paralleled by a tendency in C4 levels that nevertheless did not reach statistical significance.

*FCN2* rs76267164 (c.818G>A:p.R273Q) was detected in heterozygosis in 6 patients, 4 of which developed ARDS, from a total number of 123 informative patients. These data represent an observed frequency of 0.27% (5.26x NFE) in ARDS cases but did not exhibit significant associations with ARDS risk (**Table 2**). The rs532781899 (c.316delC:p.L117SfsX65) indel variant in the *FCN3* gene has been previously analyzed in COVID-19 cohorts and is found at a frequency of 4.1% (2.71x NFE) in our non-ARDS subcohort (**Table 2**). This frameshift variant is expected to have deleterious effects on FCN3 protein expression by introducing a premature stop codon in its Fibrinogen C-terminal domain. None of the 6 rs532781899 heterozygous carriers detected in our cohort simultaneously presented the rs76267164 polymorphism in *FCN2*.

## 4 Discussion

Since the outbreak of the SARS-CoV-2 pandemic in December 2019, great efforts have been undertaken to understand the contribution of common and rare genetic variation to COVID-19 susceptibility and severity. Complement activation has been repeatedly implicated as a critical driver of COVID-19 pathogenesis. Mouse models have shown that the initial phases of the disease are characterized by increased levels of complement components (CD55, C1r, and FH), with the classical and alternative pathways being apparently involved. Moreover, systemic complement dysregulation correlates with increased disease severity or worsened outcomes (e.g., high soluble C5b–9, FB, C3a and C5a; low FH and FI) in most cohort studies (33–35), with C5a-C5aR1 signaling playing a central pathogenic role in association with higher neutrophil extra-cellular trap formation, endothelial dysfunction, microthrombosis and ARDS (15, 36). Progression from acute to severe COVID-19 forms is characterized by an increased activation of the classical (C1r, C1s), lectin (MBL) and terminal pathways (C3, C5, C8), and the levels of C1q, C2, C4, C4b, MBL, C5, C5a, C3, C3b, FB, FD, FH and FI in plasma exhibit a significant association with disease severity (10, 37, 38). Despite the evident implication of the complement system in COVID-19 pathology, genetic associations of complement components with the disease are relatively scarce.

In the present report, we analyzed whole-exome sequencing data from 154 Spanish patients requiring hospitalization due to COVID-19 and carried out the analysis of common variants with allele frequencies ranging from 20% to 38% in NFE populations. Two polymorphisms were associated with protective effects toward ARDS: *CFHR4* rs7417769 (c.629G>A; p.N209S) and *CFH* rs1061170 (c.1204T>C; p. Y402H). Both rs7417769 and rs1061170 are located in genes belonging to the *CFH/CFHR* locus of complement regulators named after the Factor H protein (FH). FH is the main soluble modulator of C3 activation and protects self-cellular surfaces from complement attack by preventing complement activation and by accelerating the decay of the complement alternative pathway C3 convertase (C3bBb). Besides the *CFH* gene, the locus also contains the genes for five minor plasma proteins genetically and structurally related to complement factor H (*CFHR1, CFHR2, CFHR3, CFHR4 and CFHR5*) (39). FH has been previously implicated in the pathogenesis of COVID-19. On cell surfaces, FH deposition decreases SARS-CoV-2 binding and cell entry (40) by competing with the viral spike protein for binding to heparan sulfate and low FH levels are associated with complement dysregulation and severe disease (41, 42).The *CFH* polymorphism rs1061170 (p.Tyr402His) was slightly over-represented in our COVID-19 cohort (**Table 1**). This variant is in strong linkage disequilibrium with the AMD-risk FHR1*A allotype and has been previously studied in COVID-19 patients in association with reduced complement regulation and C3 overactivation, which could lead to complement-mediated uncontrolled inflammation (43, 44). In line with this observation, the significant, dose-dependent association between the rs1061170 minor allele and low IgG serum levels in our cohort, supports previous observations linking C3 consumption with reduced availability of iC3b/C3d-derived flagged antigens for antigen processing and B-cell presentation (32). Notwithstanding, we observed a substantial decreased risk of ARDS in rs1061170 carriers in our cohort (**Figure 1**), which might indicate idiosyncratic or non-complement-mediated effects of this variant on SARS-CoV2 infection but is nevertheless not supported by the literature. Similarly, the *CFHR4* rs7417769 (p.N209S) minor allele was over-represented and strongly associated with ARDS protection in the cohort (**Table 1**). In opposition to the regulatory roles of FH on C3 deposition and cleavage, FHR4 promotes complement activation by preventing Factor I-mediated C3b cleavage and is a protective factor against infection through its binding to CRP, which triggers classical pathway activation (45, 46). The rs7417769 polymorphism, which affects the fifth exon of the *CFHR4* gene, is linked to low FHR4 levels and AMD protection (25, 26). In European populations, it is frequently found within an extended haplotype (unpublished observation) that includes the aHUS-risk allele CFHR3*B (47).

The rs2230199 (p.R102G) minor (G) allele in the *C3* gene was under-represented and significantly associated with low serum C3 (**Table 1****;** **Figure 1**), in agreement with previous reports linking its minor allele with an increased risk of complement dysregulation and protection against COVID-19 infections (17). The C3F isoform, characterized by a glycine residue at position p.102 in the MG1 domain, modifies the stability of its thioester group and its capacity to react with complement-activating surfaces (8, 37). It also exhibits a weaker ability to bind to FH, which leads to reduced regulation and increased C3 consumption (17, 48).

Conversely, the rs72550870 (p.D120G) variant in the *MASP2* gene significantly correlated with high C3 levels (**Figure 1**). MASP2 deficiency, biochemically defined by plasma MASP2 levels below 100 ng/mL, affects roughly 10/10.000 of non-Finnish European individuals. Although generally asymptomatic, it has been associated with a moderate increase in infectious and autoimmune diseases (17). The rs72550870 (c.359A>G; p.D120G) variant was first described in a patient with MASP2 deficiency, autoimmunity and repeated episodes of severe pneumococcal pneumonia (28), although the direct association of the rs72550870 variant and low MASP2 levels with the patient’s phenotype has been lately considered controversial and a disease-modifying effect has been postulated instead. From 19 individuals homozygous for the rs72550870 variant identified in case-control studies, an infectious or autoimmune phenotype was detected in 8 cases (49). Considering the deleterious effects reported for this variant on MASP2 protein levels, this finding is in accordance with the prominent role attributed to the lectin pathway as an important driver of complement activation in COVID-19 (50–54). However, it has to be noted that *MASP2* results were obtained with a comparatively small number of patients (4 with ARDS versus 5 without ARDS) which restricts the robustness of the observed associations (as reflected by the wide 95% CI and non-significant p-value for ARDS-RR) and should be taken as a preliminary observation to be explored in larger cohorts.

The observed enrichment in low-complement activation variants (*CFH* rs800292 and *CFHR4* rs7417769), under-representation of the high complement activation variant *C3* rs2230199 and the association of the rs7417769 *CFHR4* variant with low FHR4 levels and ARDS protection are all consistent with the complement over-activation paradigm, according to which increased complement activation reduces infection rates and viral dissemination but are detrimental in later stages of the disease due to inflammatory complications (8).

We also analyzed the frequencies of rare variants in complement genes in the WES results and found an enrichment in six low frequency polymorphism. Two of these variants, namely rs117402032 (c.1030A>C; p.I359L) in the *C1R* gene and rs143523574 (c.100C>T; p.P34S) in the *C8A* gene, are extremely rare, both of them segregating at similar AF around 0.02% in NFE populations but exhibiting a remarkable over-representation in the COVID-19 cohort, with observed frequencies of 347x NFE and 184x NFE, respectively (**Table 2**). rs117402032 modifies a conserved isoleucine residue in the N-terminal Short Consensus Repeat (SCR) domain of C1r. The SCR domain mediates C1r interactions with C1s and C1q for the assembly and activation of the classical pathway C1 complex. Although sequence alterations in this domain can potentially modify C1r function and classical pathway activation rates, no experimental data or clinical associations are available to date. rs143523574 substitutes a conserved proline in the N-terminal end of the C8 alpha protein, near the first thrombospondin domain which is critical for MAC assembly by promoting binding and self-polymerization of C9 (53, 55).

Two of the variants identified in this study affect human Ficolin genes and potentially reduce the activation of the lectin pathway of the complement system. The *FCN2* rs76267164 (c.818G>A;p.R273Q) polymorphism exhibited a 4.75x NFE over-representation in the cohort,. It affects a highly conserved arginine in the Fibrinogen-C domain of FCN2 and is strongly associated with low FCN2 levels in plasma (*p*: 7x10–^19^ and beta coefficient: 1.36 unit decrease according to GWAS catalog accession GCST90241185 (31)). Additionally, it has been recently recognized as a genetic risk factor for the development of progressive multifocal leukoencephalopathy (PML) (32). PML is a rare brain disorder caused by the common, typically benign polyomavirus 2, also known as JC virus (JCV). In immunodepressed individuals JCV can reactivate in the brain causing severe neurological disorders with lethal consequences in a high proportion of cases.

The *FCN3* rs532781899 (c.316delC;p.L117SfsX65) variant, over-represented 1.93x NFE in the cohort (**Table 2**), was first associated with FCN3 immune deficiency in a homozygous patient with undetectable FCN3 levels, susceptibility to infections and a deficiency in FCN3–dependent complement activation (29). However the subsequent diagnosis of this patient with genetically proven Wiskott-Aldrich syndrome and later reports of FCN3-deficient individuals who were homozygous for the same variant have raised doubts about the pathogenicity of the rs532781899 variant, suggesting that, in most cases, FCN3 may be dispensable for immune defense due to redundancy in lectin pathway activation (56). At any rate, GWAS studies have linked rs532781899 to low FCN3 levels in plasma (*p* = 7x10^-186^; beta coefficient: 1.545 unit decrease, according to GWAS catalog accession GCST90247616 (30)) (**Table 3**).

The enrichment in deleterious *FCN2* and *FCN3* variants associated with low Ficolin protein levels is coherent with the proposed role of the lectin pathway as an important driver of complement activation in severe COVID-19 forms. For instance, MBL, MBL2, FCN2 and CL-11 recognition molecules bind to nucleocapsid (N) and spike (S) proteins and promote complement activation and deposition on SARS-CoV-2 (50), with plasma MASP2 concentrations significantly correlating with those of FCN2, FCN3 and the terminal complement complex (57). In the same line, the lungs and kidneys from COVID-19 deceased patients present increased deposition of MASP2, C3d and C5b-9 (52) and higher levels of lectin pathway activation products are associated with the need of oxygen support and ICU care (58). Moreover, pilot studies with the MASP-2 inhibitor Narsoplimab have shown beneficial effects in the outcome of COVID-19 patients (48, 59). Similarly, the rs4151659 (c.1693A>G: p.K565E) variant in the peptidase domain of FB was detected in 10 heterozygous patients, being similarly represented in ARDS (5.12x NFE) and non-ARDS (5.60x NFE) cases (**Table 2**), suggesting that it provides an increased risk of COVID-19 infection. This variant has been detected in AMD patients but is considered benign by protein prediction tools and there is no direct evidence that it influences FB function or complement activation.

In conclusion, our results evidence that complotype-related common polymorphisms *CFH* rs1061170 (p. Y402H) and *CFHR4* rs7417769 (p.N209S) are associated with different degrees of significant protection against ARDS while the *C3* rs2230199 (p.R102G) and *MASP2* rs72550870 (p.D120G) minor alleles are respectively associated with low and high C3 levels in COVID-19 adult patients of European descent. Due to their high population frequencies (30-40% in Non-Finnish Europeans) some of these polymorphisms constitute potential risk-stratification biomarkers. Moreover, the marked enrichment of the *C1R* rs117402032 and *C8A* rs143523574 minor alleles and increased frequencies of heterozygous carriers of alleles previously linked to reduced levels of lectin pathway activators FCN2 and FCN3 point to complement activation as a critical protective factor in the susceptibility to SARS-CoV-2 infection.

From a statistical perspective, one of the main shortcomings of the present study was the scarcity of biochemical and clinical data compared to the exome sequencing data, which was critical in restricting traceable genotype-phenotype correlations. We also note that the Non-Finnish European gnomAD frequencies we used as reference for the observed frequency ratios may differ in some cases from those listed for the Iberian population in other databases (e.g. Ensembl genome browser). However, we considered that the small Iberian cohort sizes probably do not make for a reliable representation of the allele frequencies in the Spanish population and opted for the more robust (but probably less specific) frequency data. This fact did not appreciably influence the significance of the results in **Table 1** but small deviations in the p values can eventually be detected. On the other hand, our observational design prevented case-control analyses of the polymorphic variants under study and characterization of their associated odd ratios for SARS-CoV-2 infection. Moreover, it has to be noted that the data in this study were obtained during the first wave of the pandemics (March/September 2020) and that the evolution of both pathogen strains and host acquired immunity can have a large impact on the significance of the results. However, the data presented here and specially the large over-representation of *C1R* rs117402032 C and *C8A* rs143523574 T alleles highlight the impact of common and rare genetic variation in the complement system on the susceptibility to emergent infectious disease and provide testable genetic biomarkers both for SARS-CoV-2 and future infection outbreaks.

Genetic association studies of emerging viral diseases often provide insights on the pathophysiological mechanisms driving the condition and may help identify patient groups at risk or predict disease outcomes. As such, the first infectious wave of SARS-CoV-2 during 2020, before the emergence of beta and later circulating lineages, was characterized by a relatively low number of infections compared with later stages of the pandemic, which gradually led to naturally selected, more transmissible viral variants. Both from the perspective of future SARS-CoV-2 variants but especially for other diseases eventually emerging in the future, lessons learned during the outbreak of COVID-19 may be important by drawing attention to biological mechanisms and genetic conditions conferring significant risk or protection to exposed populations.

## Data Availability

The datasets analyzed for this study can be found here: https://doi.org/10.6084/m9.figshare.32334462.
